# Place of death and associated factors in 12 Latin American countries: A total population study using death certificate data

**DOI:** 10.7189/jogh.12.04031

**Published:** 2022-04-30

**Authors:** Katja Seitz, Joachim Cohen, Luc Deliens, Andrea Cartin, Celina Castañeda de la Lanza, Emanuel A Cardozo, Fernando CI Marcucci, Leticia Viana, Luís F Rodrigues, Marvin Colorado, Victor R Samayoa, Vilma A Tripodoro, Ximena Pozo, Tania Pastrana

**Affiliations:** 1 Department of Palliative Medicine, Medical Faculty RWTH Aachen University, Aachen, Germany; 2 End-of-Life Care Research Group, Vrije Universiteit Brussel (VUB) and Ghent University, Brussels, Belgium; 3 Universidad de Costa Rica, San José, Costa Rica; 4 Coordination for Advance Directives and Palliative Care Program, Institute of Health of the State of Mexico, Ministry of Health of Mexico, Toluca, Mexico; 5 Dirección de Estadísticas en Información de Salud, National Ministry of Health, Buenos Aires, Argentina; 6 Hospital Dr. Anísio Figueiredo, State Health Secretariat of Paraná, Londrina, Brazil; 7 Department of Palliative Care and Pain, National Cancer Institute, Capiata, Paraguay; 8 Palliative Care Unit, Barreto’s Cancer Hospital, Barretos, Brazil; 9 Hospital Divina Providencia, San Salvador, El Salvador; 10 Palliative Care Unit, Institute of Cancerology, Guatemala City, Guatemala; 11 Department of Palliative Care, Institute of Medical Research A. Lanari, University of Buenos Aires, Buenos Aires, Argentina; 12 Palliative Care Unit, Hospital Comprehensive Care for the Elderly, Ministry of Public Health, Quito, Ecuador

## Abstract

**Background:**

Little is known about place of death in Latin America, although this data are crucial for health system planning. This study aims to describe place of death and associated factors in Latin America and to identify factors that contribute to inter-country differences in place of death.

**Methods:**

We conducted a total population observational study using death certificates of the total annual decedent populations in 12 countries (Argentina, Brazil, Chile, Colombia, Costa Rica, El Salvador, Guatemala, Ecuador, Mexico, Paraguay, Peru, and Uruguay) for the most recent available year (2016, 2017, or 2018). Data were analysed regarding place of death and multivariable logistic regression with place of death as the dependent variable was used to examine associated clinical and sociodemographic factors (independent variables) in each of the countries.

**Results:**

The total study sample was 2 994 685 deaths; 31.3% of deaths occurred at home, and 57.6% in hospitals. A strong variation was found among the countries with home deaths ranging from 20% (Brazil) to 67.9% (Guatemala) and hospital deaths from 22.3% (Guatemala) to 69.5% (Argentina). These differences between countries remained largely unchanged after controlling for sociodemographic factors and causes of death. The likelihood of dying at home was consistently higher with increasing age, for those living in a rural area, and for those with a lower educational level (except in Argentina).

**Conclusions:**

Most deaths in Latin America occur in hospitals, with a strong variation between countries. As clinical and sociodemographic factors included in this study did not explain country differences, other factors such as policy and health care system seem to have a crucial impact on where people die in Latin America.

Information on place of death (PoD) is crucial for informing the development of public health policies that ensure the provision of adequate end-of-life care for patients and bereaved survivors [1]. However, there is little available research about PoD in low-middle-income countries [1,2].

Studies identifying factors associated with PoD show that the determining factors for PoD are age, sex, marital status, level of education, place of residence, cause of death, and health care availability [3-8]. Since most research on this topic is based on European or North American countries, PoD patterns and their associated factors in Latin America are relatively unknown. A systematic review on PoD in Latin America performed in 2019 identified nine studies, five of which were conducted in Mexico [9-13], two in Brazil [14,15], and two in Chile [16,17]. These studies mostly focused on specific age groups or patients with cancer. Only two studies included the full population in their analysis. We found no study comparing countries in the Latin America region to each other.

The objective of this study is to describe and compare the PoD and associated factors in 12 Latin American countries using population-level death certificate (DC) data for a one-year period. The specific research questions are: What proportion of all deaths occurs at home vs in hospitals in the different Latin American countries? What factors are associated with dying at home in different countries? Which factors contribute to country differences in the proportion of deaths at home?

## METHODS

### Design

We conducted a total population observational study using death certificate (DC) data for one year from 12 countries. The study is part of the project ‘Place of death in Latin America’, which contains a database with DC data and a statistical report from 12 Spanish- and Portuguese-speaking countries in Latin America (Argentina – statistical report, Brazil, Chile, Colombia, Costa Rica, Ecuador, El Salvador, Guatemala, Mexico, Paraguay, Peru, Uruguay), representing over 90% of the Latin American population. The data sets comprise information on PoD and other relevant sociodemographic and clinical factors and refer to the years 2016, 2017, or 2018, depending on the most recent available data in the respective countries at the time of the study. A previous analysis considered the included DC data comparable (ie, containing most of the variables analysed) and of acceptable quality: high quality in three countries, medium quality in seven, and low quality in two. More details on this data collection and its resources can be found elsewhere [18].

### Population

The study population consists of all decedents aged one year and older, from 12 countries over a period of one year. We excluded subjects under one because these constitute a different type of mortality.

### Measures

For the purposes of comparison, PoD was categorized as “home”, “hospital” and “other (places)”. The sociodemographic factors included in the data file were age, sex, marital status, cause of death, level of education, and urbanization level of place of residence (rural or urban).

Marital status was unavailable in Argentina and information on level of education was unavailable in Costa Rica and El Salvador. For all deaths, the underlying cause of death was provided as International Classification of Diseases (ICD-10) code. Level of education was categorized according to the UNESCO Institute of Statistics [19], and in consultation with the local research partners was split into the levels “less than primary complete”, “primary complete”, “secondary I complete”, “secondary II complete”, “tertiary complete”.

Several ecological variables were operationalized. Urbanization levels were available for all countries except Argentina, Guatemala, Paraguay, and Peru. In El Salvador, recoding place of residence was necessary to obtain urbanization level, while in Brazil, it was made available through linkage to an external file. At country-level, potential influence of other ecological factors was estimated using data form the World Bank [20]: gross national income (GNI) per capita, hospital beds/physicians/nurses per 10 000 inhabitants, health care expenditure per capita and from the Latin American Atlas for Palliative Care [21].

### Descriptive analysis

In a descriptive analysis of the included variables, the number and percentage of deaths at home, in hospitals and in other places are described for every country along with other population characteristics (sex, place of residence in rural area, cause of death, age) which were also provided through the database. Since the database consists of population-level data, the total percentages per country database are given and no inferential statistic was calculated.

### Statistical analysis

For each country, a binary logistic regression analysis with the dependent variables “home death” vs “hospital death” was performed to identify associations between PoD and the independent variables: cause of death (cancer vs non-cancer), age, sex, marital status (excluding Argentina), and level of education (excluding Costa Rica, El Salvador). These independent variables were included due to their previous relevance in literature on PoD [3,4] and their availability through DC data. In eight countries (Brazil, Chile, Colombia, Costa Rica, El Salvador, Mexico, Peru, Uruguay) urbanization of the place of residence was available and was included in the model. The independent variables were entered simultaneously.

Additionally, a hierarchical binary logistic regression analysis with the dependent variable “home death” vs “hospital death” was performed to examine country differences in the likelihood of dying at home. Paraguay was chosen as reference country, because the percentage of home and hospital deaths were the closest to the average of all countries. Independent variables were added stepwise: country (Model 1), cause of death, age, sex, civil status (Model 2) and level of education (Model 3). Argentina, Costa Rica, and El Salvador had to be excluded from all models because of missing variables (marital status, level of education). Urbanization level of place of residence was not included in all models because criteria for categorizing areas into rural or urban varied between the countries. Independent variables were checked for multicollinearity. The hierarchical binary logistic regression analysis allows for evaluation of the extent to which observed country differences are the result of the differences in population characteristics. All cases were included in the analysis and an additional category was created for missing values (category “unknown”). To identify possible associations between independent variables caused by differences between acute and chronic illnesses, an additional subgroup analysis was conducted. In the subgroup analysis, deaths from acute heart disease and external causes were excluded.

The Pearson correlation coefficient between the percentage of home deaths and ecological factors was calculated. A correlation of >0.7 was considered high, 0.5 to 0.7 moderate, 0.3 to 0.5 low, and <0.3 as negligible [22].

All analyses were performed in IBM SPSS Statistics, version 25 (International Business Machines Corporation, Armonk NY, USA).

### Group discussion to understand the findings

We conducted a virtual group discussion with representatives and collaborators of the project. Ten participants from eight of the 12 countries invited discussed the results of the statistical analysis. The discussion was moderated by the first and last authors.

This flexible method uses a non-directive technique that results in a controlled co-production of a topic-focused discussion – in this case, the statistical analysis. It uses the group and its interactions to help participants explore and clarify their views in ways that would be less easily accessible in one-to-one interviews. The strength of group discussions lies in the “face validity” of the data generated. The collective and individual responses encouraged by the group discussion setting generate material that differs from other methods [23]. The virtual version seems to produce sufficient levels of interaction [24].

The meeting lasted 120 minutes and was recorded. Through a content analysis, explanations about how to understand the results and country differences were identified.

### Ethics

The project was approved by the Ethics committee of RWTH Aachen University (EK 206/19).

## RESULTS

### Descriptive analysis

The total number of registered deaths for persons aged 1 year and older was nearly 3 million, ranging from 22 046 in Costa Rica to 1 279 317 in Brazil, which correspond to 0.4 and 0.6% of the total population respectively. In total, 44.9% of the study population was female. Country differences were also found in age at death, with more people under 70 dying in Guatemala and over 80 in Uruguay (**Table 1**).

**Table 1:** Characteristics of deaths in 12 Latin American countries*

	Population total [20]	Death cases		Sex	Residence	CoD	Age		
**Frequency**	**Percent**	**Women**	**Rural**	**Cancer**	**1-69**	**70-79**	**≥80**
**Argentina (AR) (2017)**	44 044 811	335 109	11.2	49.0	ND	18.7	34.4	23.5	42.1
**Brazil (BR) (2017)**	207 833 825	1 279 317	42.7	44.0	15.6	17.0	48.7	21.0	30.3
**Chile (CL) (2016)**	18 209 072	102 397	3.4	47.4	13.5	25.4	35.9	23.1	41.0
**Colombia (CO) (2017)**	48 909 844	220 580	7.4	44.9	12.7	19.3	43.3	21.0	35.8
**Costa Rica (CR) (2016)**	4 899 336	22 046	0.7	43.3	23.5	22.0	43.3	19.9	36.8
**Ecuador (EC) (2017)**	16 785 356	67 487	2.3	45.0	23.5	16.3	43.7	18.9	37.3
**El Salvador (SV) (2017)**	6 388 124	38 846	1.3	43.4	37.7	8.1	49.8	18.3	31.9
**Guatemala (GT) (2017)**	16 087 418	74 100	2.5	44.2	ND	10.4	57.8	16.8	25.4
**Mexico (MX) (2017)**	124 777 326	677 591	22.6	43.9	21.6	12.4	50.2	20.0	29.8
**Paraguay (PY) (2017)**	6 867 058	27 560	0.9	43.5	ND	15.7	48.7	21.3	30.0
**Peru (PE) (2017)**	31 444 299	115 797	3.9	47.3	ND	17.0	40.2	21.2	38.6
**Uruguay (UY) (2018)**	3 449 290	33 855	1.1	49.3	4.9*	23.3	30.4	22.8	46.8
**Total**	529 695 759	2 994 685	100	44.9	17.7	16.4	46.2	20.9	32.9

ND – no data available, CoD – cause of death

*Valid data considered only (information on urbanization of place of residence missing for 71.5% of registered deaths in UY).

### Statistical analysis

#### Place of death per country

**Figure 1:**
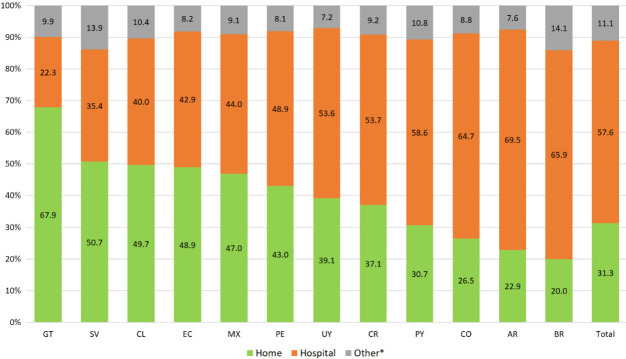
Place of death in 12 Latin American countries (n = 2 938 850), order according to percentage of home deaths. AR – Argentina, BR – Brazil, CL – Chile, CO – Colombia, CR – Costa Rica, EC – Ecuador, SV – El Salvador, GT – Guatemala, MX – Mexico, PY – Paraguay, PE – Peru, UY – Uruguay. *Includes: Public place (BR, CO, CR, SV, GT, MX, PY, PE, UY), workplace (CO, GT, PE, UY), other health care facility (BR, CO, CR, EC, SV, GT, UY), nursing home (CR), ambulance (CR), prison (UY), in transit (PE), other place (all countries).

Home death rates in Latin America ranged from 20.0% (Brazil) to 67.9% (Guatemala). The proportion of hospital deaths ranged from 22.3% (Guatemala) to 69.5% (Argentina). Information on PoD was missing in 1.9% of the cases in total. Between 7.2% (Uruguay) and 14.1% (Brazil) of deaths occurred in “other places”. Depending on the country, this category includes workplace, other health care facilities, nursing home, ambulance, prison, and other places (**Figure 1**).

#### Characteristics associated with Place of Death (per country analysis)

In all countries, probability of home death increased with older age (**Table 2**). Women were significantly less likely to die at home than men were in Argentina, Brazil, Colombia, Ecuador, Mexico, Paraguay, and Peru. The exception was Chile, where women had higher odds of dying at home than men. No significant difference between men and women emerged in other countries.

**Table 2:** Multivariable logistic regression per country with the dependent variable being death at home vs in hospital*

	AR	BR	CL	CO	CR	EC	SV	GT	MX	PY	PE	UY
**N†**	305 464	1 098 124	91 777	199 823	19 956	61 921	33 448	62 636	602 922	24 593	79 285	31 399
**Cause of death (RC: Non-cancer)**												
**Cancer**	0.78	0.61	2.83	1.27	2.93	1.33	0.79	2.73	1.787	1.42	1.61	1.14
	(0.77 to 0.80)	(0.60 to 0.62)	(2.74 to 2.93)	(1.24 to 1.30)	(2.73 to 3.14)	(1.28 to 1.39)	(0.73 to 0.85)	(2.54 to 2.93)	(1.76 to 1.82)	(1.32 to 1.53)	(1.55 to 1.68)	(1.08 to 1.21)
**Age (RC: ≥80)**												
**1-69**	0.75	0.70	0.41	0.56	0.56	0.53	0.36	0.25	0.38	0.55	0.61	0.63
	(0.73 to 0.76)	(0.69 to 0.71)	(0.40 to 0.42)	(0.55 to 0.58)	(0.52 to 0.61)	(0.51 to 0.55)	(0.34 to 0.38)	(0.24 to 0.27)	(0.37 to 0.39)	(0.51 to 0.59)	(0.58 to 0.63)	(0.60 to 0.67)
**70-79**	0.77	0.75	0.55	0.64	0.66	0.62	0.52	0.49	0.54	0.66	0.70	0.74
	(0.76 to 0.79)	(0.74 to 0.8)	(0.53 to 0.57)	(0.63 to 0.66)	(0.61 to 0.72)	(0.59 to 0.64)	(0.49 to 0.56)	(0.46 to 0.52)	(0.53 to 0.54)	(0.61 to 0.71)	(0.68 to 0.73)	(0.70 to 0.79)
**Unknown**	0.97	0.12			0.46	0.33		0.36	0.31	3.85		1.05
	(0.87 to 1.09)	(0.11 to 0.14)			(0.12 to 1.75)	(0.06 to 1.81)		(0.27 to 0.48)	(0.26 to 0.39)	(1.49 to 9.98)		(0.50 to 2.23)
**Sex (RC: Male)**												
**Female**	0.94	0.80	1.08	0.90	0.95	0.95	0.97	0.97	0.90	0.83	0.91	1.03
	(0.93 to 0.96)	(0.79 to 0.81)	(1.05 to 1.11)	(0.89 to 0.92)	(0.89 to 1.01)	(0.92 to 0.98)	(0.93 to 1.02)	(0.93 to 1.00)	(0.90 to 0.91)	(0.79 to 0.88)	(0.88 to 0.94)	(0.98 to 1.09)
**Unknown**	1.17	1.43							0.68			
	(0.97 to 1.41)	(0.93 to 2.20)							(0.28 to 1.66)			
**Marital status (RC: Married)**												
**Single**	..	1.37	1.01	1.29	0.89	1.07	1.18	0.81	1.48	1.24	0.97	1.09
		(1.35 to 1.38)	(0.98 to 1.04)	(1.26 to 1.33)	(0.82 to 0.97)	(1.03 to 1.12)	(1.12 to 1.24)	(0.78 to 0.85)	(1.46 to 1.50)	(1.17 to 1.32)	(0.94 to 1.00)	(1.02 to 1.18)
**Widowed**	..	1.13	0.93	1.18	1.17	1.03	1.08	..	1.17	1.22	1.52	1.20
		(1.12 to 1.14)	(0.89 to 0.97)	(1.14 to 1.21)	(1.07 to 1.27)	(0.98 to 1.08)	(0.98 to 1.19)		(1.16 to 1.19)	(1.12 to 1.32)	(1.45 to 1.60)	(1.12 to 1.28)
**Divorced/separated**	..	1.24	0.91	1.22	1.10	0.92	0.83	..	1.19	1.37	1.39	0.97
		(1.21 to 1.26)	(0.83 to 1.01)	(1.16 to 1.28)	(0.97 to 1.23)	(0.85 to 1.00)	(0.71 to 0.98)		(1.15 to 1.22)	(1.08 to 1.74)	(1.25 to 1.56)	(0.88 to 1.06)
**Civil partnership**	..	1.34	..	1.02	0.68	0.94	1.13	1.33	1.31	1.46	2.53	..
		(1.31 to 1.38)		(0.98 to 1.05)	(0.60 to 0.78)	(0.85 to 1.04)	(0.95 to 1.34)	(1.07 to 1.65)	(1.29 to 1.34)	(1.24 to 1.73)	(2.34 to 2.74)	
**Unknown**		1.07	1.06	0.67	1.01	0.80	0.79	0.16	0.93	0.87	1.84	0.46
		(1.04 to 1.10)	(0.93 to 1.20)	(0.64 to 0.70)	(0.80 to 1.27)	(0.71 to 0.91)	(0.69 to 0.91)	(0.12 to 0.20)	(0.90 to 0.95)	(0.73 to 1.04)	(1.71 to 1.99)	(0.43 to 0.50)
**Education level (RC: Less than primary)**												
**Primary complete**	0.82	0.86	0.95	0.76	..	0.62	..	0.47	0.87	0.67	0.70	0.87
	(0.79 to 0.85)	(0.85 to 0.87)	(0.92 to 0.99)	(0.74 to 0.78)		(0.59 to 0.65)		(0.45 to 0.49)	(0.86 to 0.89)	(0.62 to 0.71)	(0.67 to 0.72)	(0.76 to 0.98)
**Secondary I complete**	0.81	0.77	..	0.55	..	0.49	..	0.27	0.70	..	..	0.61
	(0.78 to 0.85)	(0.76 to 0.79)		(0.52 to 0.57)		(0.46 to 0.52)		(0.24 to 0.29)	(0.68 to 0.71)			(0.51 to 0.74)
**Secondary II complete**	1.33	0.87	0.94	0.75	..	0.49	..	0.25	0.68	0.49	0.58	0.46
	(1.18 to 1.49)	(0.85 to 0.89)	(0.91 to 0.98)	(0.72 to 0.79)		(0.46 to 0.53)		(0.24 to 0.27)	(0.67 to 0.70)	(0.44 to 0.54)	(0.55 to 0.60)	(0.37 to 0.57)
**Tertiary complete**	1.23	0.76	0.95	0.70	..	0.42	..	0.24	0.67	0.37	0.48	0.81
	(1.15 to 1.32)	(0.74 to 0.78)	(0.89 to 1.02)	(0.67 to 0.73)		(0.38 to 0.45)		(0.21 to 0.28)	(0.65 to 0.68)	(0.32 to 0.43)	(0.45 to 0.51)	(0.65 to 1.00)
**Unknown**	0.68	0.79	1.04	0.64		0.90		0.43	0.47	0.85	0.29	2.14
	(0.66 to 0.70)	(0.78 to 0.80)	(0.66 to 1.64)	(0.62 to 0.66)		(0.83 to 0.96)		(0.40 to 0.47)	(0.45 to 0.48)	(0.79 to 0.92)	(0.28 to 0.30)	(1.92 to 2.38)
**Urbanization (Area of residence, RC: Urban)**												
**Rural**	..	1.87	1.01	1.39	1.04	1.52	2.53	..	2.03	..	..	1.19
		(1.85 to 1.89)	(0.97 to 1.05)	(1.35 to 1.43)	(0.97 to 1.11)	(1.46 to 1.58)	(2.41 to 2.65)		(2.00 to 2.06)			(0.97 to 1.46)
**Unknown**		0.81		2.72					0.87			1.26
		(0.67 to 0.97)		(2.39 to 3.10)					(0.83 to 0.91)			(1.19 to 1.33)

RC – reference category (death in hospital), AR **–** Argentina, BR **–** Brazil, CL **–** Chile, CO **–** Colombia, CR **–** Costa Rica, EC **–** Ecuador, SV **–** El Salvador, GT **–** Guatemala, MX **–** Mexico, PY **–** Paraguay, PE **–** Peru, UY **–** Uruguay

*The odds ratio presents the likeliness for dying at home rather than in hospital for different groups of cause of death, age, sex, marital status, education level and urbanization of the area of residence compared to the reference category. The confidence intervals (95% CI) are given below the odds ratios.

†N per country may differ from the total population due to missing place of death data and excluding deaths that occurred in “other places”.

Single, as opposed to married status, was associated with a higher chance of home death in Brazil, Colombia, Ecuador, El Salvador, Mexico, Paraguay, and Uruguay, and with 11% to 19% higher odds of hospital death in Costa Rica and Guatemala, respectively (**Table 2**). Widowed, as opposed to married status, was associated with a higher chance for home death in Brazil, Colombia, Costa Rica, Mexico, Paraguay, Peru, and Uruguay, except in Chile, where the reverse was true. In El Salvador, divorced or separated people were more likely to die in a hospital, while in Brazil, Colombia, Mexico, Paraguay, and Peru home death was more likely. Civil partnership status (category “united” on the DC) was associated with greater chances for home death in Brazil, Guatemala, Mexico, Paraguay, and Peru.

Cancer conditions as underlying causes of death were associated with home (as opposed to hospital) death in all countries except for Argentina, Brazil, and El Salvador (**Table 2****).** In a subgroup analysis excluding deaths from acute heart disease and external causes, the results did not differ substantially from the main analysis. 

Except for Argentina, completion of primary or higher education (vs less than primary) was consistently associated with a lower chance for home death in all countries. The odds ratios (OR) for home death ranged from 0.47 [95% confidence interval (CI) = 0.45-0.49] in Guatemala to 0.95 (95% CI = 0.92-0.99) in Chile when having completed primary school, from 0.27 (95% CI = 0.24-0.29) in Guatemala to 0.77 (CI = 0.76-0.79) in Brazil for having completed secondary I, from 0.25 (95% CI = 0.24-0.27) in Guatemala to 0.94 (95% CI = 0.91-0.98) in Chile for having completed secondary II, and from 0.24 (95% CI = 0.21-0.28) in Guatemala to 0.76 (95% CI = 0.74-0.78) in Brazil when having completed tertiary education or higher (only statistically significant results shown). In Argentina, chances for home death were also lower for completing primary (OR = 0.82; 95% CI = 0.79-0.85) and secondary I (OR = 0.81; 95% CI = 0.78-0.85) and higher when having completed secondary II (OR = 1.33; 95% CI = 1.18-1.49) or tertiary level (OR = 1.23; 95% CI = 1.15-1.32). For this variable, the rate of missing values was particularly high in Argentina (69.2%) and Uruguay (74.5%).

In all countries providing information on urbanization levels of the place of residence, the probability of home death was higher for residents of rural rather than urban areas, except in Chile, Costa Rica, and Uruguay where the difference was not significant.

#### Multivariable analysis aimed at explaining country-variation

**Figure 2:**
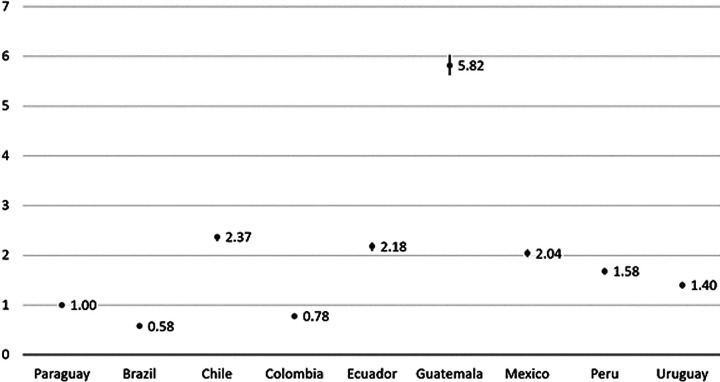
Country differences in the chances for home death vs hospital death (Odds ratios), calculated with hierarchical multivariable logistic regression with home death vs hospital death as dependent variable and country as independent variable (Model 1). Reference country: Paraguay.

Model 1 (**Figure 2**) shows the variation between countries of the likelihood of dying at home vs hospital. The closer the odds ratio is to 1 in the respective country, the closer the home death rate is to that in Paraguay (reference country). Only Brazil and Colombia had lower probabilities for home deaths than the reference country Paraguay. The probability of home death was about six times higher in Guatemala than in Paraguay. The country difference largely remained after adding sociodemographic and clinical factors to the model (Table S1, **Online Supplementary Document**).

A moderate negative correlation was observed between the proportion of home deaths and the number of available hospital beds (**Table 3**). A low negative correlation was observed between the proportion of home deaths and health care expenditure per capita as well as the number of physicians, while the GNI per capita, nursing personnel and palliative care services per 10 000 inhabitants show a negligible correlation.

**Table 3:** Country characteristics and their correlation with home deaths [20,21]

	Percentage of home deaths	GNI per capita (2018, in US$)	Healthcare expenditure per capita (2017, in US$)	Hospital beds per 10 000 inhabitants (latest year available, 2013-2015)	Medical doctors per 10 000 inhabitants (latest year available, 2016-2018)	Nursing personnel per 10 000 inhabitants (latest year available, 2016-2018)	Palliative care services located in primary care per million inhabitants (2020)
**Argentina**	22.9	12 390	1325	50	39.90	26.00	1.0
**Brazil**	20.0	9080	929	22	21.64	74.01	0.6
**Chile**	49.7	14 620	1382	22	25.91	133.25	12.7
**Colombia**	26.5	6260	459	15	21.85	13.91	0.8
**Costa Rica**	37.1	11 590	869	12	28.94	34.14	14.1
**Ecuador**	48.9	6090	518	15	20.37	25.06	2.1
**El Salvador**	50.7	3820	282	13	15.66	18.34	0.3
**Guatemala**	67.9	4390	260	6	3.55	12.83	0.2
**Mexico**	47.0	9180	495	15	23.83	23.65	0.6
**Paraguay**	30.7	5620	381	13	13.54	16.60	2.9
**Peru**	43.0	6470	333	16	13.05	29.77	0.2
**Uruguay**	39.3	15 910	1592	28	50.79	72.17	21.9
Pearson r coefficient*		-0.27	-0.34	-0.55	-0.44	-0.04	-0.01

GNI – gross national income

*Correlation with percentage of home deaths.

### Group discussion

The main findings that emerged from the group discussion were the meaning of dying at home, the lack of health care access at the end-of-life, and legal and regulatory barriers for home death (**Table 4**).

**Table 4:** Statements extracted from the group discussion about the meaning of the findings

Topic	Example
Meaning of dying at home	*Many times, those patients [that die at home] probably do not have sufficient assistance that they should have in their homes, but because of their life circumstances, they still prefer to die at home. Being able to die surrounded by their family is the biggest motive, so they ask for their discharge to die at home.* –representative from Paraguay
Health care access	*Dying at home in a rural area involves not having access to secondary or tertiary medical assistance. There are a lot of mountains and areas with limited access here, so it is very difficult in rural areas to provide primary medical care to a patient.* – representative from Guatemala
	*Those 22.9 percent [who die at home] do not necessarily receive the continuous care that they need. It seems that there are people who die in their home because they did not have any other option. Dying at home does not necessarily mean a choice, but simply being outside of the [care] system or with neglect of care.* – representative from Argentina
Legal and regulatory barriers for home death	*It is difficult to obtain the death certificate: the family must wait for hours for the person that can certify the death. So, this is a big limitation: The police officer of the closest region must come for the certification together with the treating doctor or another doctor, he has to call the prosecutor, the prosecutor has to call the coroner, and the coroner completes the death certificate. At the hospital, on the other hand, it is much simpler.* – representative from Paraguay
	*Those that can pay [for private healthcare] do not have problems. They have their own doctors or maybe private home care. In this case, they can die at home and have the death certificate guaranteed. The problem is for the public [healthcare system].* – representative from Brazil
	*Sometimes in geriatrics, immobile, bedridden patients arrive at the hospital, and I ask them why they brought them to an outpatient clinic at this stage. And they say so that when they die, you will give us the death certificate and we will have no troubles with it. And this is a sad situation. So, yes, the death certificate is a limitation, something that leads families to the hospital.* *If a person dies at home and they are part of a [healthcare] system that provides the death certificate, they call 911. There, they verify the death and call the police. The police asks if there is a death certificate. If there is no death certificate, the body is brought to forensics with the possibility of an autopsy. Everyone is scared by this, and no one wants to do this. If we ask the people, everyone wants to die at home, but the concern is: Who will give us the death certificate?* – representative from Ecuador

## DISCUSSION

We integrated total population death certificate data from 12 Latin American countries for one year into a common database with nearly 3 million deaths from 2016, 2017 or 2018. The database allowed us to investigate PoD and associated factors at the population level in Latin America. We found a strong variation between the countries with the proportion of home deaths ranging from 20% to 67.9% as well as with the proportion of hospital death from 22.3% to 69.5%. The country differences largely remained constant after adding clinical and socioeconomic factors to the model. The variation for home death of 47.9% seems to be higher than what was observed in nine Western European countries, the United States (US), Canada and New Zealand, where home death rates ranged from 13.3% in Canada up to 41.1% in Italy for patients with cancer, and from 16.1% in Canada up to 46.3% in the Netherlands for non-cancer patients [8].

Home death rates above 40% were found in Guatemala, El Salvador, Chile, Ecuador, Mexico, and Peru. In all countries except Peru, more deaths occurred at home than in hospitals in these countries. These findings show a relatively high proportion of home deaths when compared to Western Europe, the US, Canada, and New Zealand, where home death rates over 40% were observed only in Italy and among cancer patients in the Netherlands. Only cancer patients in the US and the Netherlands had more home than hospital deaths [8].

Fewer people seem to die at home in Western Europe, the US, Canada, and New Zealand than in Latin American countries. This observation does not indicate more hospitals deaths in those countries though, as a considerable amount of people die in long-term care facilities such as nursing homes [8]. Since there are few long-term care facilities in Latin America, this category was not considered relevant in our study.

### Associated factors

In most Latin American countries, home death was less likely for those with higher education levels, married people, and non-cancer patients. This points to the fact that in Latin America, people with more financial and social resources have more access to hospitals, which usually provide end-of-life care. In contrast, persons with higher education levels, and married couples have a better chance of dying at home in countries such as Italy, Belgium, the Netherlands, England, Canada, and the US [4,8]. This suggests a social gradient in PoD in both places, but in the opposite directions: Western Europeans and North Americans use scarce resources such as money, knowledge, and social connections to enable home deaths, whereas Latin Americans in many countries use those same resources to access the hospital at the end-of-life.

### Country comparison

Our analysis indicated that PoD variations between countries cannot be attributed to population characteristics or different causes of death. We explored several other possible explanations.

Although GNI does not seem a strong explanation at first, countries with higher income such as Argentina, Chile and Uruguay have higher health expenditures and can afford more hospital beds. Availability of hospital beds might contribute to variations between countries and showed a moderate negative correlation with the number of home deaths. It can explain more hospital deaths in countries with more beds per capita and more home deaths in countries with fewer beds and more limited access to health care facilities, such as El Salvador and Guatemala. However, this observation cannot be applied consistently, as we see a relevant difference in the percentage of home deaths in Ecuador (48.9%) and Colombia (26.5%), while the number of hospital beds per 10 000 inhabitants and the income per capita are similar.

Moreover, except for Chile, Costa Rica, and Uruguay, there is low availability of palliative or home care in most countries. Countries that have invested in palliative care and have comparatively more palliative care services located within in primary care report higher rates of home deaths, probably under better care, since specialized home-based palliative care services contribute to satisfaction among patients at the end-of-life [25]. Conversely, in countries that have invested little in palliative or home care services, people are often forced to organize “informal end-of-life care” outside of hospital and formal health services [26]. Private sector end-of-life care is usually only affordable for the wealthy. While a home death might mean less medical care or limited access to health care services, especially for patients with limited financial resources, families might provide care since Latin American and Caribbean cultures generally tend to value family and community-based care more than Western cultures [26,27]. A recent mortality-follow back study in Trinidad and Tobago highlighted the importance of family in the provision of palliative care [28]. Informal care may not only be a cultural norm, but also a necessity for patients dying at home in places where no other options exist. To address the lack of specialized care provision at the end-of-life in Latin America, more educational programs for generalist and specialist palliative care are needed next to implementing integrated and accessible care-providing structures [27].

There seems to be a low negative correlation between number of physicians and the percentage of home deaths, which ties in with the public investment in health, while the number of nurses was not relevant. However, this is country-level data and subnational comparisons are required to identify regions where improvement of health care availability is most needed.

In the group discussion, legal and regulatory aspects regarding the process of death certification emerged as an additional obstacle that might hinder home death. In most Latin American countries, the process is rather complicated and can, in case of a home death, require the family to involve even the police or forensics. This obligation can, in some cases, motivate people to take their family members approaching end of life to hospital, where hospital staff certify death, even if home was the preferred PoD. These bureaucratic requirements in Latin America seem to be a major obstacle to the choice of PoD and add unnecessary difficulties.

Our findings suggest that, in many parts of Latin America, PoD is defined by factors such as limited access to a hospital, which results in a lack of choice for alternatives to home as a PoD. This is different to the meaning of dying at home in many high-income countries, where it is indicative of choice and end-of-life care planning [29,30].

It is concerning that people in Latin America seem to have little access to end-of-life care (such as palliative home care services), and yet in many countries, the majority of people die at home, presumably without adequate care. End-of-life health care expenditure is especially high [31]. However, this is when patients and caregivers in Latin America are forced to provide or pay for this care themselves.

### Strengths and limitations

To our knowledge, this is the first cross-national total population study in the Latin American region to compare PoD. We integrated DC data from 12 countries with nearly 3 million total deaths into one common database and investigate where people die in Latin America and which factors are associated with PoD. Databases were obtained for 3 different years (2016, 2017, 2018), depending on the latest year available. Potential changes of death patterns within this time period were not considered relevant.

Urbanization level of place of residence (rural or urban) was not included in the cross-country comparison because of different classification criteria. In the per-country analysis, urbanization level of place of residence emerged as an important indicator for PoD and may have contributed to the explaining of the variations between countries in the final model.

The quality of DC data and the availability and completeness of the variables in the country databases is limited. In a previous quality assessment, we found the databases of El Salvador and Peru to be of low quality because in El Salvador, a high percentage of the underlying causes of death were coded to unspecific/ill-defined conditions, while in Peru, mortality registration was considered incomplete [18]. Certain variables had high rates of missing values: PoD was missing in 25.5% in Peru. Education level was missing in 74.5% of the registered deaths in Uruguay, in 69.2% in Argentina and in between 19.3% to 26.9% in Brazil, Colombia, Paraguay, and Peru. In Uruguay, urbanization of the area of residence was missing in 71.5%. In Colombia, Peru, and Uruguay, marital status was missing in 13.2%, 10.5% and 15.1% respectively. In the multivariate regression, Argentina, Costa Rica, and El Salvador were excluded because of missing variables and are thus not reflected in the cross-country comparison. However, the remaining amount of important data of good quality allowed the analysis. In order to decrease the impact of data quality (such as a high number of missing values), we adjusted the statistical models by adding a separate category for missing values within the given variables. In many cases, the OR for this category was not significant. It must be taken into account that the quality of death certificate data largely depends on how accurate the certificate was completed by the certifying person. More training for medical staff on how to complete death certificates and creating more awareness for the relevance of death certification could improve death certificate data and death registration in general. For cross-country comparisons, more homogenous categories of place of death and other variables could be a way to improve the comparability across the Latin American region. There are further variables possibly relevant for country differences, for instance the quality of medical diagnostics and treatment. We tried to reflect this through a correlation analysis including indicators such as ‘health care expenditure or medical doctors per 10 000 inhabitants’ (**Table 3**). As this seems to have an impact on place of death, further research is needed to explore the associations not only on a cross-country level, but also on a subnational level.

The online group discussion was limited by the number of participating countries (only 8 of the 12 included countries represented).

## CONCLUSIONS

Although most deaths in Latin America occur in hospitals, the number of hospital and home deaths vary greatly between the countries. Death at home is associated with older age, living in a rural area, marital status, and education level. The factors included in our analysis could not explain the variations between countries. This suggests that other factors such as health system resources, availability of care, policy and legislation have a crucial impact on where people die.

Further research is needed to explore regional differences within countries and the circumstances of home and hospital deaths, since they are difficult to compare to Europe or North America. For the improvement of end-of-life care in Latin America, further analyses focusing on a population with chronic illness, where end-of-life care can be planned, can shed light on availability for the population in need of these services.

## Additional material


Online Supplementary Document
