# Erratum: Seitz K, Cohen J, Deliens L, Cartin A, Castañeda de la Lanza C, Cardozo EA, Marcucci FC, Viana L, Rodrigues LF, Colorado M, Samayoa VR, Tripodoro VA, Pozo X, Pastrana T. Place of death and associated factors in 12 Latin American countries: A total population study using death certificate data. J Glob Health. 2022 Apr 30;12:04031.

**DOI:** 10.7189/jogh.14.1204031err1

**Published:** 2024-09-27

**Authors:** Journal of Global Health Editorial Team



Following the publication of the manuscript ‘Place of death and associated factors in 12 Latin American countries: A total population study using death certificate data’ on 30 April 2022, the authors noted that they incorrectly labelled two rows in Table 2 and introduced several errors into the manuscript text, as follows:

Table 2, third row: ‘Cause of death (RC: Cancer)’ should have been labelled as ‘Cause of death (RC: Non-cancer)’.

Table 2, fourth row: ‘Non-cancer’ should be ‘cancer’.

‘Results’ section, subsection ‘Characteristics associated with Place of Death (per country analysis)’, third paragraph: ‘Non-cancer conditions as underlying causes of death were associated with home (as opposed to hospital) death in all countries except for Argentina, Brazil, and El Salvador (Table 2)’ should read ‘Cancer conditions as underlying causes of death were associated with home (as opposed to hospital) death in all countries except for Argentina, Brazil, and El Salvador (Table 2)’.

‘Discussion’ section, subsection ‘Associated factors’, first paragraph: ‘In most Latin American countries, home death was less likely for those with higher education levels, married people, and cancer patients’ should read as ‘In most Latin American countries, home death was less likely for those with higher education levels, married people, and non-cancer patients’

‘Discussion’ section, subsection ‘Associated factors’, first paragraph: ‘In contrast, persons with higher education levels, married couples, and cancer patients have a better chance of dying at home in countries such as Italy, Belgium, the Netherlands, England, Canada, and the US [4,8]’ should read ‘In contrast, persons with higher education levels and married couples have a better chance of dying at home in countries such as Italy, Belgium, the Netherlands, England, Canada, and the US [4,8]’

The authors regret these errors and apologise for any inconvenience they may have caused.

These corrections have been made to the online and the PDF version of the manuscript on 27 September 2024, following the authors’ notice on 15 May 2024.

