# Supplementary material for: Place of death and associated factors in 12 Latin American countries: A total population study using death certificate data
Source: J Glob Health. 2022 Apr 30;12:04031. doi: 10.7189/jogh.12.04031 (PMC9078151; doi:10.7189/jogh.12.04031)
Supplement: Online Supplementary Document [file jogh-12-04031-s001.pdf]

Table S1: Country differences in the chances for home death vs. hospital death

	Model 1	Model 2	Model 3
Paraguay	1	1	1
Brazil	0.58 (0.57-0.60)	0.58 (0.57-0.60)	0.58 (0.57-0.60)
Chile	2.37 (2.30-2.44)	2.31 (2.24-2.38)	2.22 (2.15-2.29)
Colombia	0,78 (0.76-0.81)	0.76 (0.74-0.78)	0.80 (0.78-0.82)
Ecuador	2.18 (2.11-2.24)	2.12 (2.05-2.19)	2.23 (2.16-2.30)
Guatemala	5.82 (5.64-6.01)	6.17 (5.97-6.37)	5.79 (5.61-5.98)
Mexico	2.04 (1.99-2,10)	2.15 (2.09-2.20)	2.12 (2.06-2.18)
Peru	1.68 (1.63-1.73)	1.64 (1.59-1.69)	1.68 (1.63-1.73)
Uruguay	1.40 (1.35-1.45)	1.34 (1.30-1.39)	1.56 (1.50-1.61)

Odds ratios were calculated with hierarchical multivariable logistic regression with home death vs. hospital death as dependent variable. Independent variables: Model 1: country. Model 2: country, cause of death, age, sex, marital status. Model 3: country, cause of death, age, sex, marital status, education level. Reference country: Paraguay.
